# Permeation of 2-Butoxyethanol Through Multiple Layers of a Disposable Nitrile Glove Material and a Single Layer of Microflex 93-260

**DOI:** 10.3390/ma18215055

**Published:** 2025-11-06

**Authors:** Eun Jin Song Kuramoto, Shane Que Hee

**Affiliations:** Department of Environmental Health Sciences, Fielding School of Public Health, University of California Los Angeles, 650 Charles Young Jr. Drive South, Los Angeles, CA 90095-1772, USA; eunjinppo@ucla.edu

**Keywords:** 2-butoxyethanol, disposable nitrile gloves, double and triple gloving, Microflex 93-260, permeation

## Abstract

Double gloving of disposable gloves occurs in healthcare when extra protection is required against carcinogens, sensitizers, pathogens and sharps. Triple gloving is much rarer. The resistances of single, double and triple layers of disposable nitrile glove material against 2-butoxyethanol (2-BE) were compared with the resistance of a single layer of Microflex 93-260 (Microflex). Three 2.54 cm ASTM F739 permeation cells with closed-loop water collection without recirculation in a moving tray water bath at 35.0 ± 0.5 °C facilitated the permeation relative to a blank cell. Capillary gas chromatography/mass spectrometry allowed the determination of the standardized breakthrough time (SBT), steady state permeation rate (SSPR) and cumulated permeated mass/area (CPM/A) by 30 min. Two nitrile layers (267 ± 14 µm) were about the same thickness as the Microflex layer (249 ± 6 µm). Statistical analysis showed equivalence at *p* ≤ 0.05 of the multiple layers and the Microflex layer relative to average SBT and CPM/A by 30 min, all such comparisons with the single nitrile layer also being statistically different. The triple layer had an average SSPR or post-breakthrough permeation rate 8 times lower than its single layer, while that for the Microflex layer was 1.5 times lower. Thus, the Microflex layer in terms of CPM/A by 30 min at 210 ± 40 µg/cm^2^ appeared closer to two nitrile layers (520 ± 560 µg/cm^2^) than three (93 ± 93 µg/cm^2^).

## 1. Introduction

Gloves are made of materials designed to resist hazardous physical, chemical, biological and mechanical stressors primarily exposing the skin of the hands [1]. In 2019 before the COVID-19 lockdown, there were approximately 18,200 skin-related illnesses recorded across all industries in the United States, about 1.7 times those for inhalation [2].

Many skin exposures occur through the handling of chemicals for which chemically protective clothing (CPC) gloves should be worn as personal protective equipment (PPE) [3,4]. To assess material barrier resistance, permeation (mass transfer through the material at the molecular level) and penetration (mass transfer through nano- or micro- holes and seams) tests are performed [3,4]. Most researchers and glove manufacturers in the United States have used the gas-collection mode of ASTM F739 [5] in its open- and closed-loop versions to generate data from circular glove pieces. Methods that use liquid collection for nonvolatile chemicals are valid alternatives provided the permeant is adequately soluble, the solvent does not degrade or back-permeate the glove material, and the collection side is mixed. Water is usually the preferred collection solvent. The major permeation parameters measured at 27 ± 1 °C over eight hours or less and sampled at 5 min intervals are the standardized breakthrough time (SBT) defined at 100 ng/cm^2^/min for open- and closed-loop collection systems and the steady state permeation rate (SSPR) where the permeation rate is maximal and constant.

Disposable gloves that permit manipulation of small items are extensively used in healthcare and clinical chemistry laboratories [3,4]. The major permeation protocol there, ASTM D6978-19 [6], tests pieces of gloves in the large ASTM-F739 cell where circular glove pieces exposed to aqueous solutions of specific chemotherapy agents at their highest use concentrations are sampled at 30 min intervals over four hours from a mixed aqueous closed-loop collection system without recirculation. The ASTM D6978-19 method differs from ASTM F739 in that permeations are at 35 ± 1 °C; a permeation rate of 10 ng/cm^2^/min defines the breakthrough detection time; and the thinnest portion of the glove from either the cuff or the palm is evaluated rather than the palm or top of the palm for ASTM F739.

Similar types of cells and procedures are mandated for glove testing for occupational environments within the European Community through the EN 374 and ISO 6529 methods (now combined) [3], with their past respective normalized breakthrough times at 1000 and 100 ng/cm^2^/min being analogous to the ASTM F739 SBT. Recent reviews [1,3,4] have discussed how the resistance results from the ASTM F739 and other permeation cell methods relate to actual glove protection in the workplace, where stretching, abrasion, flexing and sweating, as well as sudden temperature and relative humidity changes, occur.

Double gloving with disposable gloves has been practiced in healthcare industries to protect workers. Research on this usually involves aqueous challenges, double layering of circular glove pieces in permeation cells to simulate field double gloving, and closed-loop aqueous collection systems without recirculation [3,4]. The first peer-reviewed journal report of double layering of disposable nitrile glove material against a pure organic solvent, diethylene glycol mono-n-butyl ether, was published in 2020, and also featured a review of the double gloving literature in its introduction [7]. Double layers from four different disposable nitrile gloves from one company caused average normalized breakthrough times at 250 ng/cm^2^/5 min to be lengthened about fourfold and average SSPR to be lowered by about the same factor with percent relative standard deviations (RSDs) for each of about ±20%. Since then, more reports have appeared on double gloving against toxic drugs like antineoplastics [8,9]. Double gloving in pandemics [10] and in surgeries [11,12,13] has also been reviewed.

Triple gloving of disposable gloves is a much rarer technique employed by surgeons and dentists mostly for puncture protection to resist potent body fluid pathogens [14,15,16,17,18] like the Ebola virus [17] and COVID-19 [18], and to protect against dental sensitizers such as acrylates [19]. Firefighters at HazMat incidents also have triple gloving [20]. There are no permeation cell literature reports of quantitative comparisons of permeation of pure organic solvents through single, double and triple layers of disposable gloves of the same or different glove material.

Suitable compounds for testing must not degrade the glove [3,4]. Disposable gloves are designed to resist water H-O-H. The closest organic molecules to water are alcohols, R-O-H, where R is alkyl, alicyclyl, aryl or other non-polar entity. However, methanol, ethanol and n-butanol permeate the Kimberly-Clark Professional disposable Stirling Nitrile glove in 10 min or less at ASTM F739 conditions [21]. This implies that higher carbon content alcohols would be better choices. 2-Butoxyethanol (2-BE; n-butyl-O-CH_2_CH_2_-OH; CAS RN 111-76-2; ethylene glycol mono-n-butyl ether, butyl cellosolve and butyl oxitol are among the many synonyms) is a water-miscible solvent with a 168 °C boiling point [22]. It is used in paints and varnishes, household and healthcare facility soaps, cleaning liquids and detergents, pesticide formulations (as active ingredient and adjuvant), floor wax stripping agents, fracking, textile processing and dry-cleaning, as well as in adhesives, cosmetics and oil slick dispersants [22].

There are several air concentration guidelines in U.S. workplaces for 2-BE. The Occupational Safety and Health Administration (OSHA) permissible exposure limit (PEL) regulation is 50 ppm (skin) [23]. The American Conference of Governmental Industrial Hygienists (ACGIH) recommends an 8 h threshold limit value/time weighted average (TLV-TWA) of 20 ppm (*v*/*v*) associated with eye and upper respiratory tract effects, and a urinary Biological Exposure Index (BEI) of post-shift total 200 mg 2-butoxyacetic acid/g creatinine [24]. The National Institute for Occupational Safety and Health (NIOSH) recommended exposure limit (REL) is 5 ppm (skin), and its immediately dangerous to life or health level is 700 ppm [23].

Ansell documented for 2-BE the following “Excellent” degradation and “Good Protection” ASTM F739 SBTs of its thinnest CPC gloves [25]: >480 min for Butyl ChemTek 38-514 (14 mil or 360 µm), LLDPE Barrier 02-100 (2.5 mil or 64 µm), Neoprene 39-865 (18 mil or 460 µm), and Viton Butyl ChemTek 38-612 (12 mil or 310 µm); 240–480 min for nitrile Solvex 37-145 (11 mil or 280 µm) and nitrile AlphaTek 58-435 (18 mil or 460 µm). Butyl, neoprene and nitrile had the longest SBTs of the non-laminates/blends. Nitrile was chosen to be the disposable glove material investigated in terms of multilayers since it is the most used and available disposable glove material [26]. The thickest powderless unsupported and unlined disposable nitrile glove of Kimtech Science was Blue (130 µm thickness or about 5.1 mil from previous research [7]). Ansell in the latter half of the 2010s introduced its “thinnest chemical-resistant disposable glove”, the Microflex 93-260 (Microtex), 7.8 mil (200 µm) thick, comprising three layers of nitrile and neoprene (polychloroprene) [27,28]. The Microflex glove is also ASTM D6978 compliant for chemotherapy drugs. Since butyl and chloroprene CPC gloves were more resistant to 2-BE than Solvex CPC nitrile relative to SBT [25], the trilayered Microflex glove was tested relative to the disposable nitrile layers since the resistances to 2-BE for both glove types were unknown.

The hypothesis was that three layers of disposable nitrile would produce ASTM F739 SBTs not statistically different from one layer of Microflex. Thus, 2-BE was to be used in an ASTM F739 permeation cell to investigate permeation of single, double and triple layers of the thickest Kimberly-Clark disposable nitrile glove, Kimtech Science Blue, and to compare with the results from Ansell’s Microflex.

## 2. Materials and Methods

### 2.1. Glove Selection

The gloves used were unsupported, unlined and powderless Kimberly-Clark Professional’s Kimtech Science Blue nitrile disposable gloves from Fisher Scientific, Pittsburgh, PA, USA, and Microflex 93-260 nitrile/neoprene gloves from Ansell, Iselin, NJ, USA.

### 2.2. Chemicals

2-BE (99%) was procured from Eastman Chemical Company, Miami, FL, USA. 4-Bromophenol internal standard (IS) was obtained from Aldrich, St Louis, MO, USA. Sodium dichromate (99%) was used for a saturated aqueous solution to generate (54 ± 4)% relative humidity inside Pyrex glass vacuum desiccators, all from Fisher Scientific, Pittsburgh, PA, USA.

Water for aqueous solutions was obtained from a Millipore Milli-Q Water System (Temecula, CA, USA) and Millipore Simplicity Water Purification final polishing system (Temecula, CA, USA). Helium (99.9999%) and nitrogen (99.9999%) were purchased from Air Liquide (El Segundo, CA, USA).

### 2.3. Equipment

Two sampling-side 2.54 cm ASTM-type-I-PTC-600 permeation cells from Pesce Lab Sales (Kennett Square, PA, USA) were used in permeation testing. Fisher Scientific (Pittsburgh, PA, USA) was the source for a Marathon digital micrometer to measure glove palm-piece thickness at three random locations; vernier calipers to determine glove piece diameters; a moving tray Model 2870 shaking water bath for temperature control and mixing; a torque wrench to tighten permeation cell nuts uniformly at 5 ft-lbs; and a calibrated traceable printing hygrometer/thermometer for measurement of relative humidity and temperature.

The gas chromatograph/mass spectrometer (GC-MS) system for analyses was an Agilent (Santa Clara, CA, USA) 6890N Network GC with a 30 m × 0.25 mm HP-5 ms chemically bonded (0.25 μm thick film) fused silica capillary column in tandem with a quadrupole mass spectrometer, the Agilent 5973 Network Mass Selective Detector, operated at 70 eV electron impact energy at an ion source temperature of 230 °C. The GC-MS transfer line and GC injector temperatures were 280 °C. Helium was the carrier gas at 3.0 ± 0.1 mL/min.

Microscopic magnification of the glove surface with an American Optical MicroStar hand-held microscope (Buffalo, NY, USA) was used to determine whether microholes or tears were present before and after permeation.

### 2.4. Permeation Testing

The ASTM F739 test protocol with the permeation cells in the closed loop without recirculation mode [5] was modified: the temperature was 35.0 ± 0.4 °C from ASTM D6978-19 [6]; two challenge half-cells were utilized instead of the challenge/collection half-cells; and sampling was performed without collection solvent replenishment.

Test specimens were cut with scissors from the palm areas of gloves previously conditioned at 54 ± 3% relative humidity at 25 ± 1 °C for 24 h. The test pieces were checked for microholes and their thicknesses measured.

**Figure 1:**
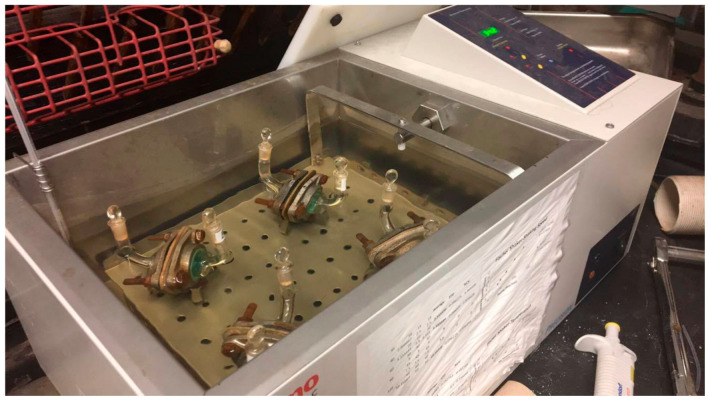
The permeation cell setup in the thermostatted shaking water bath at zero permeation time [29].

The test piece(s) with outer surface(s) facing the challenge chamber were then mounted between the PTFE gaskets, sealed by the flanges, and the nuts tightened. For gloving simulations, two pieces were used instead of one for double gloving, and three for triple gloving. The four assembled cells that were immersed except for the top halves of their stems in the water bath (Figure 1) were maintained at a shaking speed of 8.52 ± 0.05 cm/s to eliminate concentration gradients in the collection solvent. At the start of the 30 min equilibration period at 35 °C, 10.0 mL of triple de-ionized water was added to the four collection sides and any leaking noted. The test chemical (10.0 mL) was added at permeation time zero to the three challenge cells and the fourth cell acted as the air method blank. The mixing was restarted. Collection side sampling occurred at 0.0, 5, 10, 20, 30, 40, 50, 60, 120 and 135 min. The samples were taken with a 100 µL Eppendorf pipet with long tips (moving tray stopped), placed into pre-chilled 1 mL Pyrex vials and then stored at −20 °C. The glove samples were reconditioned at the original humidity conditions before the remeasuring of all the initial parameters.

### 2.5. Analyte Analysis

The samples were thawed and aliquots injected for GC-MS quantitation were 2.0 μL in volume containing 0.10 µg/µL of IS. The MS ions had a mass-to-charge ratio (*m*/*z*) of 57 and *m*/*z* of 87 for 2-BE, and *m*/*z* 172 for the IS. There was a solvent delay of 4 min at 120 °C, held for 2 min, ramped at 40 °C/min to 280 °C and kept there for 4 min.

GC-MS quantitations were obtained by the internal standard method whereby the area response of analyte injected divided by the area of the IS was interpolated on a linear plot of that ratio for standards versus mass of analyte injected. The standard concentrations were 0, 0.1, 0.5, 1, 5, 10, 50 and 100 ng/µL for the low range and 100, 200, 300 and 400 ng/µL for the high range. The linear portions were characterized by their slopes, intercepts, their associated standard deviations, the correlation coefficients r and *p*-values. For the permeation samples, dilution into a working linear range with water solvent occurred when necessary.

The analyte mass in the collection side was calculated by multiplying the injected sample mass by the permeation cell collection solvent volume in μL at the sampling time divided by two. The total mass collected in the collection side (corrected for mass removed by previous collection) divided by the exposed surface area was then plotted versus sampling time from zero time in min to generate the permeation curves for each individual glove tested. The sampling time interval where the permeation reached 100 ng/cm^2^/min was determined to be the SBT. The time period of steepest slope was identified as the steady state permeation period and its slope (the SSPR) and standard deviation obtained through linear regression. The lag time t_l_ was obtained from this linear regression equation for the time when the cumulated mass divided by exposed area was zero. The diffusion coefficient D_p_ was then calculated from Equation (1) [3]:

D_p_ = l^2^/6t_l_ (1)

where l is the initial thickness in cm, t_l_ is the lag time in min and D_p_ is in cm^2^/min.

The mean and standard deviation data for each triplicate set for each glove type were then calculated.

### 2.6. Statistical Analyses

Linear regression was used to characterize linear relationships, including standard deviations of the slopes and intercepts as well as defining the correlation coefficients r and *p*-value for GC-MS IS analysis data that involved area analyte/IS ratios versus injected analyte mass. The *Student t*-test was used to test the statistical significance of differences in arithmetic means for all triplicate permeation experimental data, for example, thickness for each glove before and after permeation for swelling determination, and average SBT, cumulated permeated mass/area at 30 min, SSPR and D_p_ for each glove type. The threshold *p*-value was *p* ≤ 0.05. These parameters were obtained with Microsoft Excel 2007 software.

## 3. Results

### 3.1. Analyte Analyses

The retention times for the 2-BE and the 4-BP were 4.8 min and 7.9 min, respectively. The total run time for each injection was 10 min. The regression equation for the low concentration standards (0, 0.1, 0.5, 1, 5, 10, 50 and 100 ng/µL) was y = 0.0553x + 0.0126 (r^2^ = 0.9904, *p* ≤ 0.05). The lower quantifiable limit was 0.10 ng/µL. The regression equation for the high concentration standards range (100, 200, 300 and 400 ng/µL) was y = 0.7058x − 40.9 (r^2^ = 0.9985, *p* ≤ 0.05).

### 3.2. Glove Thickness

Table 1 shows all the intrarun and interrun data for glove thickness and swelling, SBT, SSPR and calculated D_p_.

Microscopic examination revealed no obvious microholes in the materials before and after permeation, confirming that penetration did not occur. Relative to interrun initial glove thickness, the imprecision data in terms of % relative standard deviation (RSD) of layers were as follows: 14, single nitrile layer; 6.2, double nitrile layer; 3.3, triple nitrile layer; and 2.7, Microflex single layer. The after-permeation respective data were 19, 5.2, 4.7 and 2.4. The respective percent differences between before and after challenge thickness were 43, 11, 12 and 12 with their RSDs ≥ 46%. In terms of initial thickness, the Microflex glove was approximately equivalent to a double layer of the disposable nitrile glove, which was not statistically different at *p* ≤ 0.05.

The intrarun thickness change was swelling in all but the one instance for a single nitrile layer. The resolution of the Marathon digital micrometer was 1 µm, and hence that of thickness differences was 2 µm. The percent swelling ranges and their span are instructive: single nitrile, −6.3 to 9.3, 15.6; double nitrile, 6 to 14, 8; triple nitrile, 4 to 21, 17; and Microflex, 9.2 to 13, 3.8. The Microflex layer is clearly more uniformly affected by 2-BE than all the nitrile layers, probably because of better quality assurance and control in the production process than for the disposable nitrile materials.

### 3.3. Standardized Breakthrough Time

When the midpoints of the triplicate sampling intervals that contained the SBT were averaged for each layering condition (Table 1), the average SBTs for the nitrile glove were 4.2 ± 2.9 min (RSD, 69%) for the single layer; 13 ± 4 min (RSD, 31%) for the double layer; and 18 ± 13 min (RSD, 72%) for the triple layer. The comparison of the triple layer with the double resulted in no statistical significance at *p* ≤ 0.05 because of large standard deviations but comparison of single-/double-layer and single-/triple-layer nitrile data were significant at *p* ≤ 0.05.

The Microflex layer had an average SBT of 22 ± 12 min (RSD, 55%). In terms of average SBT, the Microflex layer behaved like the double and triple nitrile layers since they were not statistically different at *p* ≤ 0.05, though the average SBT for double nitrile layers was 3.1 times longer than that for a single nitrile layer, 4.3 times longer for triple nitrile layers and 5.2 times longer than the single Microflex layer. Based just on average SBT and ignoring standard deviations, the Microflex layer was most resistant to 2-BE.

### 3.4. Cumulated Permeated Mass/Area

Another permeation resistance parameter related to risk assessment is cumulated permeated mass per unit area CPM/A (µg/cm^2^) at critical times. By 30 min, this parameter average was single layer nitrile, 1040 ± 280 (RSD, 27%); double, 520 ± 560 (RSD, 108%); triple, 93 ± 93 (RSD, 100%); and Microflex, 210 ± 40 (RSD, 19%). While the only statistically significant pair comparisons at *p* ≤ 0.05 were between the triple layer and the two single layers and also between the two single layers, the clear implication is that the triple layer is about 2.3 times more resistant to 2-BE at 30 min than Microflex, and both are much more resistant than a single nitrile layer (about 11 times for three nitrile layers and 5 times for Microflex). The Microflex layer provided resistance intermediate between two and three nitrile layers.

### 3.5. Steady State Permeation Rate

The average SSPR for the nitrile layers in μg/cm^2^/min was (Table 1) 42 ± 9 (RSD, 21%) for the single layer; 50 ± 60 (RSD, 120%) for the double layer; and 5 ± 5 (RSD, 100%) for the triple layer, the respective SSPR relative to the single layer being not significant for the double layer and about eight times lower for the triple layer, the latter significantly different at *p* ≤ 0.05. The value for the triple layer may not be the true SSPR but there is no doubt the permeation rate is much lower than the other layers. The Microflex layer had an average SSPR of 29 ± 14 μg/cm^2^/min (RSD, 48%), about 1.4 times lower than for a single nitrile layer but 6 times higher than the triple layer. All other comparisons were not statistically significant at *p* ≤ 0.05 because of the large imprecisions.

### 3.6. Diffusion Coefficient

The calculated D_p_ values from equation 1 are presented in Table 1 but are placed there for reference because of the high degree of intrarun swelling reported in Section 3.2. A swelling classification for individual gloves is available [30]: none (not statistically significant), moderate (about 4%) and complete (10–20%). When there is no swelling, Fick’s first law of diffusion can be used to interpret the permeation data and to calculate diffusion coefficients from equation 1. None of the average diffusion coefficients for nitrile layers differed from one another because of high imprecision.

## 4. Discussion

The data are consistent with a strong interaction between the glove test materials and 2-BE resulting in high RSDs for all the interrun data for swelling, SBTs, CPM/Asat 30 min and SSPRs. In a previous study with 2-BE permeating through a single layer of Kimtech Science Purple nitrile disposable glove (101 ± 1 µm thickness) under the same experimental conditions as the present study’s, the SSPR had an RSD of 21% and the CPM/Aat 20 min was 6.6 ± 2.1 µg/cm^2^ with an RSD of 32% [31], comparable with the RSD data in the present study. SBTs for that purple nitrile layer were between 0 and 20 min but are not comparable with the present single nitrile layer SBT results because a 20 min testing interval was used. The average swelling was only 1.9 ± 1.9%, with an RSD of 100%.

Though the physical thickness of the Microflex layer most resembled two nitrile layers, its permeation behavior was closer to three nitrile layers, and generally exhibited the lowest RSDs. In terms of the CPM/A results for the Microflex and triple nitrile layers, the greater resistance of the nitrile triple layer can be explained by the Microflex layer having the longer SBT but also a higher SSPR or post-breakthrough permeation rate than the triple nitrile layer so that how long a glove is worn becomes important. Disposable gloves are not meant to be worn for long periods, unlike CPC gloves, where SSPR assumes greater importance but is still largely ignored, as evident in the Kimberly-Clark Worldwide/Kimtech Science/Ansell classification [21]. Its permeation ASTM F739 SBTs in min for their gloves are as follows: Not Recommended, <10; Splash Protection, 10–60; Medium Protection, 60–240; High Protection, >240. These are also subdivided into levels in min: Unclassified <10; 1, 10–30; 2, 30–60; 3, 60–120; 4, 120–240; 5, 240–480; 6, >480. Thus, the average SBTs in Table 1 would be classified as Not Recommended for one nitrile layer and Splash Protection for the other situations. This classification does not discriminate between the SBTs of the multiple nitrile layers and those of the Microflex layer. The level classification allows better resolution and the best splash protection is shown by the Microflex layer, but even at 30 min SSPR or the permeation rate before the steady state period may be important relative to the potential dose to the hands and therefore health effects. In terms of the hypothesis, a triple or double nitrile layer can replace the Microflex layer in terms of SBTs for these gloves and 2-BE under tightly controlled laboratory conditions. The same may not be so for different chemicals and gloves, and much more research is needed in the laboratory and workplace.

These are the first permeation cell data published on triple layers of disposable glove materials for a pure organic solvent challenge simulating triple gloving. A similar study for the simulation of double gloving of disposable nitrile gloves for a pure organic solvent challenge has been previously reported [7].

The use of double or triple gloving with disposable gloves may be the only at-hand PPE recourse in emergencies when CPC gloves are unavailable. When donning multiple gloves, the most protective glove should be first [32] and the smallest size that fits last, to optimize manipulations with small implements or items to be handled [33]. If only one type of glove is available in different thicknesses, the thickest should be first and the thinnest last.

It is hoped that more layer research with different and differing disposable gloves and challenge chemicals will be conducted based on our approach to find more practical means to ensure safety during emergencies when CPC gloves may not be available but disposable gloves are.

## 5. Conclusions

This is the first peer-reviewed journal article that quantitatively compares the permeation of a pure organic chemical, 2-butoxyethanol, through single, double and triple layers of the same glove material in a permeation cell.

The hypothesis was proven that the standardized breakthrough time at 100 ng/cm^2^/min for three layers of blue disposable nitrile glove material using a modified ASTM F739 protocol at 35 °C would be as resistant as a single layer of the Ansell nitrile/neoprene trilaminate Microflex 93-260, though two layers also sufficed. The permeation parameter, cumulated permeated mass per unit area at 30 min, produced the same results.

More research with different and the same glove materials and chemicals is needed to guide disposable glove selection when chemical protective clothing gloves are absent.

Field glove studies with workers under workplace conditions should occur to confirm the permeation cell screening data.

## Figures and Tables

**Table 1:** Thickness (A = initial µm; B = after permeation µm; C = B-A µm; D = change %), standardized breakthrough time (SBT), steady state permeation rate (SSPR) and calculated diffusion coefficient (D_p_) of 2-butoxyethanol through layers of a Kimtech Science Blue disposable nitrile glove (N) and a single layer of Microflex 93-260 (M) in an ASTM F739 2.54 cm permeation cell with water collection without recirculation at 35 °C. Standard deviations (SDs) in parentheses are for triplicate data.

Layer	Replicate	Thickness				SBT Interval	SSPR	D_p_ × 10^6^
#	#	A	B	C	D	/Midpoint, min	μg/cm^2^/min	cm^2^/min
N1	1	128	140	12	9.3	0–5/2.5	34.4	5.61
	2	160	172	12	7.5	5–10/7.5	40.6	6.93
	3	127	119	−8	−6.3	0–5/2.5	51.7	13.9
	Mean (SD)	138 (19)	144 (27)	6 (46)	4.3 (33)	0–10/4.2 (2.9)	42 (9)	9 (5)
N2	1	223	255	32	14	10–20/15	12.7	2.86
	2	249	264	15	6.0	5–10/7.5	20.3	16.8
	3	250	282	32	13	10–20/15	113	5.23
	Mean (SD)	241 (15)	267 (14)	26 (29)	11 (12)	5–20/13 (4)	49 (56)	8 (8)
N3	1	356	429	73	21	20–30/25	0.270	10.8
	2	361	399	38	11	0–5/2.5	6.60	59.7
	3	378	393	15	4.0	20–30/25	9.30	7.85
	Mean (SD)	365 (12)	407 (19)	42 (31)	12 (8.9)	0–30/18 (13)	5.4 (4.6)	30 (30)
M1	1	228	249	21	9.2	10–20/15	18.5	4.39
	2	216	243	27	13	10–20/15	24.7	4.00
	3	226	254	28	12	30–40/35	44.7	2.32
	Mean (SD)	223 (6)	249 (6)	26 (12)	12 (5.5)	10–40/22 (12)	29 (14)	4 (1)

## Data Availability

The original contributions presented in this study are included in the article. Further inquiries can be directed to the corresponding author.
